# Affordability, Feasibility, and Accessibility: Companion Animal Guardians with (Dis)Abilities’ Access to Veterinary Medical and Behavioral Services during COVID-19

**DOI:** 10.3390/ani11082359

**Published:** 2021-08-10

**Authors:** Haorui Wu, Ravinder Sarah Bains, Amy Morris, Celeste Morales

**Affiliations:** 1 School of Social Work, Dalhousie University, Halifax, NS B3H 4R2, Canada; rv569663@dal.ca; 2 Natural Hazards Center, University of Colorado Boulder, Boulder, CO 80309, USA; 3 Vancouver Humane Society, Vancouver, BC V6P 5A2, Canada; amy@vancouverhumanesociety.bc.ca (A.M.); celeste@vancouverhumanesociety.bc.ca (C.M.)

**Keywords:** COVID-19, PWDs, companion animals, companion animal guardians, veterinary services

## Abstract

**Simple Summary:**

Compounding pre-existing diverse vulnerabilities, pet owners living with (dis)abilities are expected to confront more challenges than their peers without (dis)abilities. However, there is a paucity of literature investigating COVID-19-specific impacts on access to veterinary medical and behavioral services from the lens of persons with (dis)abilities (PWDs). Through semi-structured in-depth interviews, this study highlights PWDs’ difficulties, three-fold: (1) COVID-19 has worsened PWDs’ already precarious financial capacity to pay for veterinary services, (2) existing assistance and support programs do not effectively address PWDs’ unique requirements to support their companion animals’ health needs, and (3) public health protocols triggered public transportation challenges for PWDs who must now also adapt to complicated curbside services. Building PWD-driven social assistance and support programs would help reduce these challenges and promote health and well-being for both PWDs and their companion animals.

**Abstract:**

The research aims to explore COVID-19 health and safety protocol impacts on companion animal guardians living with (dis)abilities relating to veterinary medical and behavioral service access. The COVID-19 global public health crisis has impacted almost all international communities; however, vulnerable and marginalized groups have been disproportionately affected. Within the human–companion animal domain, COVID-19-driven societal impacts (e.g., social, health, and economic) not only boomed with new companion animal guardians, but also negatively influenced guardians’ access to veterinary services. Although studies have examined guardian-related COVID-19-specific challenges, there is a paucity of concentration on vulnerable populations, such as persons with disabilities (PWDs). Responding to this research deficit, this study recruited twelve companion animal guardians to participate in semi-structured in-depth interviews, and eight (67%) of the twelve participants self-identified as PWDs. From a PWD perspective, this research reveals three pandemic-triggered primary barriers, preventing PWDs from pursuing veterinary services: (1) service affordability, (2) assistance program feasibility, and (3) veterinary service accessibility. This article argues that PWD-driven approaches could improve existing assistance and support programs to address PWDs’ unique requirements, promoting a healthy human–animal bond.

## 1. Introduction

The global public health emergency of COVID-19 has had catastrophic influences on human settlements worldwide; however, vulnerable and marginalized populations have been disproportionately affected, including older people [1], gender and racial or ethnic minorities [2], people experiencing homelessness [3], and persons with (dis)abilities (PWDs) [4]. Out of these traditional domains, current studies have continually identified nascent vulnerable groups, for example, companion animal guardians [5,6], who experienced COVID-19-specific hardships. Combining the traditional and nascent groups, there is a paucity of literature that explores the challenges that companion animal guardians with (dis)abilities have been confronted with within the current global pandemic context.

When the first wave of COVID-19 triggered a worldwide lockdown, only essential services were permitted to maintain the societal operation of critical functions [7]. In response to public health protocols, most veterinary hospitals/clinics adjusted their service delivery models to focus on essential care only [8]. Furthermore, the stay-at-home order also triggered the breaking of the 20-year record in booming companion animal guardianship, generating a surge in demand for veterinary medical and behavioral services [9]. Declined veterinary services and increased service demands jointly exacerbated companion animal guardians’ access to veterinary medicinal and behavioral services [10]. Although researchers have quantitatively explored companion animal guardians’ veterinary service access challenges during COVID-19 [7], an in-depth examination of these challenges associated with companion animal guardians’ pre-COVID-19 existing vulnerabilities (e.g., financial status, access to social assistance programs and veterinary services) remains under-researched. Consequently, from the PWDs’ perspective, this study qualitatively addresses the challenges companion animal guardians faced in their pursuit of veterinary medical and behavioral interventions during the first wave of COVID-19 in Metro Vancouver Regional District (henceforward, Metro Vancouver), British Columbia (BC), Canada.

## 2. PWDs and Access to Veterinary Services during the COVID-19 Pandemic

Generally, animals have been increasingly considered “loved family members,” bringing tremendous benefits to their guardians and families [11]. These benefits have been widely and essentially identified within PWD–animal interactions [12,13]. The global public health emergency of COVID-19 has significantly impacted public and veterinary services, which support both PWD well-being and animal welfare. Utilizing social services and assistance for PWDs, and veterinary services, the following section portrays a conceptual framework to identify the existing research deficits associated with COVID-19-driven influences.

### 2.1. PWDs: Compounded Challenges during the COVID-19 Pandemic

In addition to health- and well-being-related challenges (e.g., chronic illness, mental health, low overall well-being) [14,15], PWDs struggle with various difficulties, socially [16], culturally [17], and politically [18]. In particular, rapid assessment of (dis)ability surveys in India illustrates that lack of information and transport are the top two barriers that prevent PWDs from accessing different public services and assistance programs [19]. Furthermore, economic challenges have long-term negative impacts on PWDs [20]. Indeed, in 2014, the low-income rate of PWDs, who constitute 20% of the total Canadian population aged 25 to 64, was more than 2.5 times greater than those without a (dis)ability [21]. The COVID-19-specific factors (e.g., unemployment and isolation) have further aggravated the negative impacts of economics. To be precise, the probability of COVID-19-driven financial hardship, which lower earnings groups experienced, is at least two times higher than the top quintile earners [22]. The reduced public services and protective measures seriously decreased PWDs’ accessibility towards health and social services [23]. The compounding influences of various barriers exacerbate the existing vulnerable status of PWDs and their extended family, especially service and companion animals.

Current research has significantly contributed to the examination of diverse health and well-being to identify the relevance and benefits that service animals provided for their guardians with (dis)abilities across their entire life span [24,25,26]. Legally, service animals trained for special needs are viewed as equipment necessary for PWDs to manage certain types of daily tasks [27]. Although companion animals (pets) are not considered service animals [28], companion animals enhance their guardians’ physical health, emotional wellness, and overall well-being [29]. Hence, it is vital to acknowledge the various health and well-being benefits which companion animals provide to their guardians. Past studies have predominantly focused on human benefits within the human–companion animal bond [16]. Assisting companion animals to access veterinary medical and behavioral services, although gaining attention, remains under-researched, especially in disaster settings (e.g., hurricane and pandemic). For example, in the global context of the COVID-19 pandemic, community-based veterinary service assistance programs (low-cost or even free) enhance the veterinary experiences for disadvantaged companion animal guardians affected by the pandemic [30]. Moreover, research has discovered that advancing pet attachment, financial status, and household composition would prevent pet relinquishment and contribute to a healthy human–animal bond [31]. These studies utilized a quantitative approach to survey the general public. Research focusing on a particular vulnerable group, PWD as an example, remains inadequate.

Extreme events (e.g., hurricanes, earthquakes, and pandemics) impact both humans and more-than-human communities [7]. The literature on disaster and emergency management strongly suggests including companion animals in family-based pre-disaster planning [5]. Companion animals rely on their guardians to maintain their care and well-being [32]. The sudden and swift unfoldment of COVID-19 led to companion animal guardians feeling fear, anxiety, and concern with their capacity to provide veterinary services for their animals [33]. The compounding hardships towards PWDs, from pandemic and non-disaster scenarios, have reshaped companion animal guardians’ attitudes towards their animals’ veterinary services. Although the literature explores various challenges PWDs confront in diverse disaster settings, there is a gap in how these hardships alter PWDs’ behaviors relating to veterinary service access [34].

### 2.2. Social Service and Assistance: Unclear Impacts on PWDs with Companion Animals

Government and non-government organizations worldwide offer public service and assistance targeting various societal vulnerabilities, promoting an equal, just, and inclusive society [35]. The regular government assistance programs for PWDs aim to guarantee basic living requirements while barely providing adequate shelter and living expenses, whereas additional costs such as animal care are not taken into consideration [36]. As a response to the current pandemic, like other countries worldwide, the Canadian Emergency Response Benefit (CERB) provides financial support for affected individuals. However, PWDs are constantly challenged when seeking sustainable and gainful employment on account of the individual’s physical and/or mental abilities, lack of inclusive employers, and/or modifications in the workplace [7]. Hence, most PWDs were not eligible for CERB because their previous income did not meet the benchmark. It has been argued that COVID-19 has worsened Canadian financial support, trapping PWDs in an uncertain economic future and accelerating wealth inequality [37]. In addition, it affects PWDs’ ability to provide timely veterinary services for their companion animals.

Most nonprofit organizations, which offer social service and assistance programs, rely on government funding and charitable donations. The COVID-19-driven economic downturn has weakened these organizations’ capacity to continue their programs [38,39]. In addition, animal assistance programs have limited capacity to assist families on a long-term basis due to parameters that cap aid at once per family. For example, in BC, Canada, animal protection programs witnessed a dramatic decrease in food and supply donations and volunteers [40]. Additionally, public health protocols have paused physical and in-person fundraising activities for these community-based animal protection agencies, posing social and economic barriers. Furthermore, unique PWD challenges, including mobility, hearing, and cognition, present additional hurdles to obtaining information regarding social services and assistance [41]. COVID-19-driven social isolation further impacts the PWDs’ information acquisition. In brief, the time-sensitive issue regarding how reduced social service and assistance programs impact PWDs’ access to veterinary services has not been deeply explored.

### 2.3. Adaptive Veterinary Service: Lack of Evaluation from PWDs

During the first wave of the lockdown, although veterinary medical and behavioral interventions were recognized as essential services in provinces across Canada, only urgent care was allowed initially, and later, curbside service was permitted [42]. Additionally, COVID-19-specific precautionary measures created delays in veterinary clinic supply delivery, while increasing service demand from diminished clinic service capacity [43]. Many studies focused on ethically challenging situations encountered in a veterinary clinic context, such as what is deemed essential, conflicts between the interests of clients and the interests of their animals, and how to proceed when clientele have limited finances [44,45,46,47]. However, studies were lacking in investigating how these unique situations impact companion animal guardians who are vulnerable and marginalized PWDs.

Adaptive veterinary service during COVID-19 stressed frontline veterinary professionals. Research demonstrates that veterinary technicians experienced compassion fatigue and burnout relating to stress and low compensation while navigating the unknown territory of virus transmission to animals [48]. The alternative service models of curbside service and telemedicine avoid potential virus transmission [49] and benefit the trainer–vet relationship [50]. Virtual communication might not be accessible for PWDs, especially those posed with physical and cognitive challenges [51,52]. However, there is a lack of literature to examine the adaptive veterinary service from companion animal guardians’ perspective, especially those living with physical and mental health challenges.

Succinctly, COVID-19-driven difficulties and weaknesses in animal care during the pandemic have led to increased concerns, hardships, and ultimately stress [5]. These challenges cannot be seen in isolation from the societal landscape. Examining these challenges from the three domains above (PWDs, reduced social services and assistance, and adaptive veterinary service) further indicates complicities and interconnections. Research deficits associated with these complicities and interconnections reveal a comprehensive understanding of pandemic-related impacts to veterinary service access in PWDs [53]. Furthermore, the research will contribute to in-depth, evidence-based knowledge of this marginalized population’s experiences of owning animals during the pandemic. From a PWD perspective, this article is centered on the question: what are the challenges companion animal guardians face in their pursuit of veterinary medical and behavioral interventions during COVID-19?

## 3. Materials and Methods

This qualitative study employed a phenomenological lens to contribute to a nuanced understanding of companion animal guardians’ challenges regarding access to veterinary medical and behavioral services during the first wave of COVID-19. Utilizing an interpretivist paradigm [54], this study was grounded in the assumption that the companion animal guardians’ attitudes and behaviors of obtaining veterinary services were negatively reshaped by COVID-19-driven societal consequences. Accordingly, a purposive sampling strategy [55] was used to recruit 12 COVID-19 affected companion animal guardians, who applied and received companion animal veterinary emergency funds offered by the Vancouver Humane Society (VHS). These 12 participants were invited for individual, in-depth, semi-structured interviews. The semi-structured interview allows freedom for both the interviewer and the interviewee to address emerging topics during the discussions [56,57]. This study was funded by the Social Sciences and Humanities Research Ethics Board at Dalhousie University (certificate number: 2020-5371).

### 3.1. Settings

The 12 participants lived in Metro Vancouver, BC, Canada, during the first wave of COVID-19. This study focused on urban community residents because the rural communities in BC have had long-term accessibility challenges towards veterinary services prior to COVID-19 [58]. Furthermore, Metro Vancouver is the most densely populated geographic region, with over 50% of the provincial population [59]. It is the third most populated urban area across Canada according to the 2016 national census [60]. This metropolitan region has been severely affected by COVID-19 since January 2020, with the first confirmed case identified on 28 January 2020 [61] and reporting over 80% of the province’s confirmed cases of COVID-19 [42,62]. These backgrounds establish a platform to comprehensively examine the companion animal guardians’ attitudes and behaviors regarding accessing veterinary services during the first wave of COVID-19.

### 3.2. Participants

The research team randomly contacted 29 applicants who obtained the VHS’s companion animal veterinary emergency funds. The first 12 participants who were able to provide participation consent were invited for a telephone interview. The number of participants reached the benchmark for qualitative content analysis [62]. Verbal consent was obtained at the beginning of each interview. The demographic factor of (dis)ability was neither among the inclusion/exclusion criteria nor identified through interview questions, however, among the 12 participants, 8 participants self-identified as PWDs. The demographic information of the participants is shown in Table 1. Although (dis)ability was not the original intent of this study, the prevalence of self-identification of living with disabilities, emerging as a special theme during data analysis (see below), stimulates the conceptualization of this article from the perspective of PWDs.

### 3.3. Interviews

The research team developed an interview protocol. Following the interview protocol, the 12 interviews were conducted by 2 team members, a sociologist (10 interviews) and a social worker (2 interviews), from December 2020 to May 2021. The median duration of each interview was approximately 45 minutes. Open-ended questions were asked during the discussion through the following five major sections: (1) companion animal guardians’ basic information (e.g., demographic information and their companion animals), (2) COVID-19-driven challenges, (3) resources and support to address these challenges, (4) suggestions for veterinary service and animal protection agencies, and (5) lessons learned and suggestions for other companion animal guardians affected by pandemics or other extreme events. Under each section, detailed interview questions were asked. The interviewers adjusted the consequence of interview questions according to the participants’ responses. The research protocol (including all interview questions) can be accessed through a disaster-specific open-access data repository, Designsafe-Cyberinfrastructure (please see details in [63]). The interviews were audio-recorded, transcribed, and analyzed by qualitative data analysis software NVivo 12 (QSR International, Melbourne, Australia).

### 3.4. Data Analysis

A content analysis approach was utilized to analyze the interview transcripts [64]. Two research team members, with expertise in disaster and emergency management and disability social work respectively, coded all the interview transcripts independently and collaboratively elaborated themes through two rounds of data analysis, namely deductive (top-down) and inductive (bottom-up) strategies [65]. Combining both deductive and inductive strategies guarantees inter-rater consistency when the two members code the same data [66].

In particular, although (dis)ability was not included among the participant recruitment inclusion/exclusion criteria, (dis)ability emerged as one of the nine primary categories after the first round of deductive analysis. Other primary categories consist of, for example, financial barriers, information resources, and veterinary service providers. The two analysts elaborated their coding process and recognized the strong and unique interconnections between (dis)abilities and the eight other categories, preventing the companion animal guardians from accessing veterinary medical and behavioral services [67,68]. Drawing on these logical and substantiated ties, the second round of data analysis used the bottom-up (inductive) approach to explore how the eight primary categories associate with the participants’ (dis)ability status, in order to further identify the fundamental factors that reshaped these participants’ attitudes. The second round of data analysis mainly focused on the narratives of the eight participants who self-identified as PDWs. The narratives of the four participants who did not self-identify with (dis)abilities provided supporting information to comparatively evaluate and elaborate on the challenges that participants living with (dis)abilities indicated.

**Figure 1:**
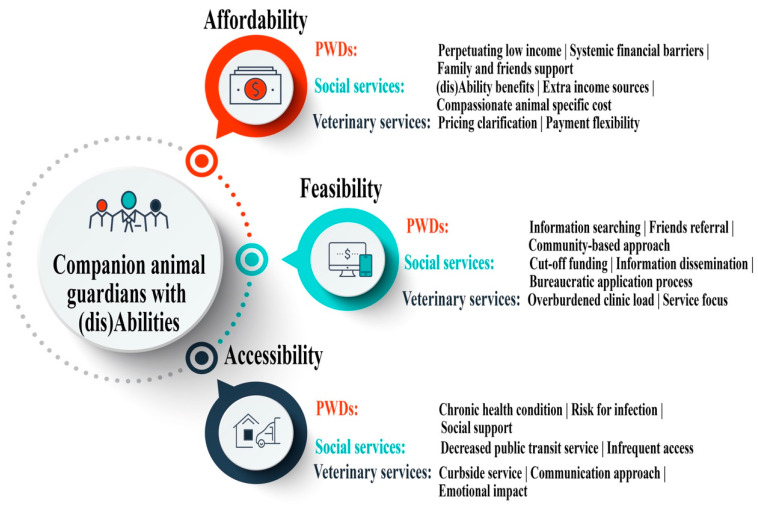
Themes and codes. The three circles (Affordability, Feasibility, and Accessibility) demonstrate the three themes that emerged in the data analysis regarding PWDs’ access to veterinary services during the first lockdown of COVID-19. Under each theme, tri-level sub-categories were developed to provide detailed supportive information, namely PWD (RED), social service (GREEN), and veterinary service (NAVY). Each subtheme was followed by different codes, which were used to identify related information from interview transcripts.

The deductive approach encouraged the researchers to broadly identify diverse COVID-19-driven factors, directly and indirectly impacting the participants’ attitudes and behaviors regarding veterinary services. The inductive approach enabled the researchers to retrospectively examine all the logical and substantiated ties between living with (dis)abilities and the COVID-19-driven factors. Integrating both deductive and inductive strategies empowers the researchers to apply the lens of (dis)ability to comprehensively explore this unique group’s access to veterinary services during the first wave of COVID-19. As shown in Figure 1, three main (dis)ability-related themes (affordability, feasibility, and accessibility) were evolved. Three sub-categories were created, namely individuals, public service in general, and veterinary service in particular. Each sub-category was supported by various codes (listed after each sub-category).

## 4. Results

This section reports the PWD-specific themes, affordability (for veterinary medical and behavioral service), feasibility (to access-related assistance programs), and accessibility (to transport to veterinary hospitals/clinics). These themes chronically portray different stages inextricably linked to companion animal guardians’ challenges, concerns, and barriers at the three levels of individual, social service, and veterinary service.

### 4.1. Affordability for Veterinary Medical and Behavioral Services

#### 4.1.1. At the Companion Animal Guardian Level—Perpetuating and Worsened Financial Status

As discussed previously, people with (dis)abilities subsist on at least 2.5 times lower income than their non-(dis)abled peers [23]. Like other vulnerable and marginalized populations, the pandemic has intensified their ongoing low economic status, directly jeopardizing the affordability of their companion animal’s medical and behavioral needs.

One participant, a self-employed musician who originally had a complete list of performances arranged for 2020, bespoke that: “last year (2019) was going to be like a record year for gigs. I was really excited…However, all of a sudden, all the gigs booked right up until the end of the year (2020) all got canceled.” The cancellation directly impeded this participant’s current and prospective financial fluidity, further hindering their capacity to pay for veterinary bills that may arise. To access supplemental income, another participant mentioned that “[Before COVID-19] I go out collecting empty bottles once a week to help buy some extra groceries [including pet food] and put gas in the vehicle.” The COVID-19-driven public health protocols, particularly the “stay at home” order, had limited the bottle collection activities, further reducing the complementary income.

The perpetuating low income is a barrier for fulfilling living requirements, let alone veterinary services. Participants highlighted that their limited payments and benefits did not allocate enough space for the veterinary bills. Specifically, one participant described her reaction to noticing the amount of a veterinary bill: “The cost of $900. I just dropped to my knees. I didn’t know what to do.” Hence, participants denoted various approaches for securing financial support through their personal networks and broader social connections. As an illustration, some participants chose to approach a few different family members and friends for loans; however, the pandemic also affected these potential lenders’ financial capacity. One participant explained that in order to pay for emergency veterinary medical care, she created a loan “combined with about four friends.” Furthermore, obtaining loans has the propensity to challenge the relationships between the recipients and their loaners, and negative impacts can transpire on their emotional well-being, particularly when they cannot repay on time. Nonetheless, the commitment to their companion animals propelled them to opt for this risky option.

#### 4.1.2. At the Social Service Level: Neglecting the Companion Animal Guardians’ Unique Needs

In Canada, the government-designated social protection programs (e.g., basic income plan) are the principal or even the only income for most PWDs [69]. For example, BC residents with (dis)abilities can apply for Person with Disabilities status, with a monthly payment of $1350.42 for a single individual [59]. Individuals must prepare long, complicated documents, which can be a challenge for those with limited executive functioning, along with other mandates, such as strict eligibility criteria and a physician report (which can be challenging to provide if the (dis)ability is ‘invisible’). Even if successful, the monthly allotments may not be enough to support a single person’s basic living requirements in Metro Vancouver, the most expensive place to live in Canada [70]. Moreover, extra medicine and rehabilitation costs, which are very common among PWDs, would increase these people’s financial burden [71].

One participant mentioned that: “(dis)ability doesn’t give you that much money. My rent is almost all of that [(dis)ability benefits]. You know, for quite a few years, I’ve had to make money on the side just to be able to feed my cats.”

Since governmental support does not provide a bridge to their financial gap, in order to avoid succumbing to economic difficulties, some individuals attain access to much-needed social services to fulfill their basic needs, such as food banks and clothing donations.

One participant felt very guilty and expressed that: “[During the lockdown] I could still get food [for my dog and me] from food banks. I prayed every day that my dog wouldn’t get sick because I cannot pay for that. I felt terrible when I saw my dog was sick because I could not do anything.”

Companion animals provide various benefits and support towards PWDs within different age categories [72,73]. Both examples speak volumes about the barriers associated with the existing social assistance programs, which might not address these companion animal guardians’ specific requirements, such as incurring extra expenses for their companion animals and rectifying a perpetuating poverty issue. Since PWDs present a higher level of mental health issues than their peers [74], the second example has demonstrated that being unable to pay for their companion animal’s veterinary medical service had generated extra emotional burdens. These barriers have led to unfavorable influences on the health and well-being of both companion animals and their guardians.

#### 4.1.3. At the Veterinary Service Level—Pricing Clarification and Payment Flexibility

The COVID-19 lockdown has dramatically reduced veterinary clinics’ in-hospital patient intake capacity and service hours, moving veterinary professional–client meetings to a virtual setting [75]. Several participants narrated that they might have to check different veterinary clinics nearby to identify the most suitable service. During this searching process, pricing played a critical role in final decision making due to their current vulnerable financial status.

One participant described that “my favorite vet was not able to take my cat. I first googled the [animal hospitals] around my neighborhood and checked their reviews and pricing. [However], the pricing information is not available on their websites. Then I called [each one].”

“My vet used to show me a list of treatments and walked through them one by one!” another participant recalled. Since face-to-face communication was not available, some participants revealed that they felt confused while discussing treatment plans by phone with veterinary professionals. Some participants’ (dis)abilities pose barriers to virtual communication. Furthermore, variances in veterinarian-proposed treatment plans resulted in price quotation discrepancies. During virtual communication, price quotations might not be addressed in full detail, triggering further confusion. Another participant argued that some COVID-19-driven charges might not be reasonable. “I phoned the emergency vet, there was a $75 exam fee, and a $75 emergency fee, but you are an emergency vet so why are you charging an emergency fee in the first place?”

After selecting their ideal veterinary clinics, the participants revealed that negotiating payment flexibility was not permitted, even if they claimed their vulnerabilities. One participant suggested that veterinary clinics “should be more forgiving on asking for, you know, like an $800 deposit.” Moreover, the lack of payment flexibility continues to uphold the oppression PWDs encounter where their bravery to begin advocating for their needs goes unheard. Hence, some participants proposed that veterinary clinics could consider the clientele’s unique circumstances and provide possible payment flexibility during the pandemic, in particular for people with (dis)abilities. One participant indicated that “[payment flexibility] could release a lot of pressure. It is really helpful for people who are suffering from mental health issues like me.”

### 4.2. Feasibility—To Obtain Related Assistance Programs

#### 4.2.1. Individuals—Obtaining Assistance Program Information

Reduced affordability encourages companion animal guardians to pursue various assistance programs, especially those offered by local animal protection organizations (e.g., a humane society). Web-based searches for these community-based agencies are a commonly used approach. After successfully securing some financial support from a local organization, one participant provided some suggestions: “[companion animal guardians with financial difficulties] should search online and check your local humane society. There might be some assistant programs for you.”

However, this commonly used approach might not be feasible for everyone, especially those living with (dis)abilities. Some participants shared that they struggle to pay for basic living needs. Their financial status cannot permit a budget for cell phone service, Internet access, and costly technological devices. Additionally, physical and/or cognitive challenges may lead to experiencing difficulties in navigating search engines and social media platforms, hindering the individual’s ability to seek relevant information. These diverse challenges merged to jeopardize the participants’ information feasibility, resulting in unawareness of existing assistance programs. One participant stated that “I didn’t even know that [animal assistance programs] existed or was an option.”

Companion animal guardians’ social networks also provided some reliable information sources. One participant mentioned that she obtained the assistance program information from other companion animal guardians who were connected by walking their dogs: “I wish more people knew about services that are out there and to ask.” Accordingly, one participant posted her cat’s emergency illness on social media: “I received a list of all the organizations which could provide help!” Another participant shared her unsuccessful experience “I did not know what was wrong with my cat. I put flyers [in the community center] with a picture of my cat.” Notably, however, during the pandemic, community centers were closed or operating with reduced service hours, so her posted request for assistance did not reach the intended audience.

Focusing on individual information pursing strategies, these examples indicate the three critical channels for companion animal guardians to seek out information, including individual (Internet search), neighborhood (connected with other companion animal guardians), and community (community centers). Although compounding factors associated with their (dis)abilities and the pandemic produced extra challenges, these three channels suggest the potential pathways to spread awareness about the assistance programs.

#### 4.2.2. Public Services—Assistance Programs and Information Circulation

The COVID-19-specific economic influence also reduced the nonprofit organizations’ capacity of offering animal assistance programs. Participants reportedly approached different organizations inquiring about assistance. One participant shared: “I think in general there [is] like not enough [animal assistance] programs, especially in Metro Vancouver.” Another participant expressed frustration when phoning different agencies, and discovered the continued response was “we don’t have funds right now.”

If funding was available yet an emergency arose, the administrative process such as applications and processing times might not support the uncontrollable circumstance. One participant highlighted, “if someone needs like funding, I think you know, it’s kind of hard. If you’re in an emergency that you’d have to go apply, and if it’s a week and no one will get back to you right away there, I would say there needs to be help in the emergency aspect.”

As stated in the section above, PWDs might have challenges accessing various information sources. One participant expressed that “services should be widely known.” The wide circulation of related information is critical, not only to help (dis)abled companion animal guardians and others who have the same challenges, but also to advance the service agencies’ mission. In addition to traditional information dissemination channels (e.g., websites and public media), service agencies’ networks and partnerships would support the information circulation.

One participant with mental health issues provided some suggestions: “I need to see a counselor frequently. It would be great if [my mental health clinic] has the information. People [with (dis)abilities] always need different assistance, [these places] could be good [to circulate the information].” Other participants indicated that the service agencies could collaborate with veterinary clinics so that these assistance programs would be more visible.

#### 4.2.3. Veterinary Service Organizations—Overburdened Clinic Activities

The record-breaking worldwide boom in companion animal guardianship during the pandemic has caused a surge in demand for veterinary medical and behavioral services [76]. This surge, plus the shortage of human and physical resources, resulted in overburdened animal hospitals/clinics, with most of them capable of only focusing on maintaining their basic services [77]. The College of Veterinarians of BC provides information about practice facilities pertaining to health inspections and medical records [78]. However, referrals for assistance programs are not included. Hence, promoting assistance program information might not be considered by most clinics during this challenging period.

One participant described: “I got [the assistance program information] from SPCA (Society for the Prevention of Cruelty to Animals). I do not think other [regular] veterinary hospitals would provide that.” Most veterinary clinics are for-profit organizations. Promoting social services might not be their primary focus, and doing so might add extra responsibilities to veterinary professionals’ already overwhelmed working routine. This further impacts the PWDs’ feasibility to obtain information independently.

### 4.3. Accessibility—Transporting to Veterinary Hospitals/Clinics and Adapting to Curbside Service

#### 4.3.1. Companion Animal Guardians—Health-Related Concerns

The global pandemic stimulated the development of precautionary measures to support the health and safety of the general public. The Government of Canada implemented safety measures (e.g., wearing masks and keeping social distance) in public spaces. PWDs may experience chronic health conditions which correlate to decreased immune systems. During the pandemic, accessing safe public services triggered deep individual concerns associated with physical health and mental wellness, presenting potential access challenges to veterinary medical and behavioral services. This section addresses these challenges and relative solutions at the individual level.

Participants highlighted difficulties in commuting to veterinary clinics due to the constant fear of contracting COVID-19. For instance, one immunocompromised participant indicated the stress of accessing transportation: “I was afraid to take a cab because I have three autoimmune diseases, right?” Furthermore, during transportation, participants mentioned they had to endure a stressful commute to the clinic by managing their companion animals’ unfavorable behaviors (e.g., being loud, hissing, and constantly moving), which they found arduous. One participant shared that holding their companion animal in a taxi cab “when my cat is screaming like I’m ticking him off” is unsustainable for a prolonged distance. These two examples demonstrate that COVID-19 has created an ongoing struggle within their complex lives, emphasizing another layer of challenges.

This accessibility factor triggered the participants to pursue help from their friends and families. Specifically, participants mentioned that connecting with their friends to obtain transportation made them feel that “someone was willing to help,” “they were cared for,” and “they were not alone in their situation.” One participant appreciated her friend’s accompaniment while waiting in the car outside of the clinic using the curbside service: “I was so worried and did not know what was happening. My friend held my hands and told me everything would be fine.” These cases demonstrated the various benefits of receiving support from social connections. On the other hand, without relevant support, some companion animal guardians might be prevented from pursuing veterinary services.

#### 4.3.2. Public Services—Physical Health Risks in Using Public Transit

Metro Vancouver has the best and largest public transportation system (e.g., bus and Skytrain) in North America, and the public transportation system is the first choice or even the only choice for most residents [79]. Responding to the COVID-19-driven public health safety measures, public transportation reduced operational hours, frequency, and passenger capacity [80]. This reduced service resulted in long waiting times and tiresome routes for companion animal guardians to commute with companion animals to veterinary clinics. One participant indicated that the one-way travel to reach the veterinary clinic destination increased from approximately 20 min to “almost an hour.” Moreover, as PWDs may experience physical impairments, standing or sitting during commutes for extended periods may aggravate their chronic pain. Despite Metro Vancouver’s public transit system allowing companion animals onboard, strict rules must be followed (e.g., companion animals must be kept in small, clean, odor-free enclosed cages rather than exposed) [81]. Travel with companion animals is suggested during off-peak hours, yet this public service may not coincide with veterinary appointments.

As most participants in this study access PWD funding, a monthly $52.00 transportation supplement is allocated to each recipient, supporting their accessibilities toward utilizing public transit [59]. Although accessibility designs have improved PWDs’ access towards public transportation, there are (dis)ability-related factors that might prevent people with (dis)abilities from taking public transportation, including physical or cognitive barriers to holding a bus pass in their hands, distributing cash into the fare collector, and problem-solving mathematical calculations to determine the remaining funds balance in their transportation account [82,83]. Notably, PWDs may face a greater risk of experiencing severe COVID-19 symptoms due to a high prevalence of comorbidity [83]. Furthermore, the inter-transmission of COVID-19 between human and companion animals is still under investigation [84]. Therefore, taking companion animals into public transportation may increase the exposure of PWDs, their animals, and other passengers towards risk, especially when community spread has been identified.

Briefly, public services have developed relative strategies to fulfill diverse requirements, especially for vulnerable persons. The COVID-19 pandemic triggered extra barriers for PWDs. Their (dis)ability-related challenges, compounded with the pandemic-specific issues (e.g., reduced service time and physical health concerns), inform a complex circumstance, ultimately decreasing the capacity for PWDs to fluidly access transportation.

#### 4.3.3. Veterinary Services—Adapting to Curbside Service

From telemedicine (remote consultations to clients) to emergency calls only, to curbside service, veterinary clinics adopted effective strategies to mitigate the spread of COVID-19, protecting veterinary professionals, companion animals, and their guardians. This modified service model led to participants expressing the emotional impacts associated with accessing veterinary services, primarily regarding their inability to be close to their companion animals and waiting to hear about their clinical progress, demonstrating undue stress.

One participant described their experience while waiting in a parking lot with other companion animal guardians: “nobody could go in with their pets, and you could see people like stressing out because there were lots of us there. Everybody was stressing out in the parking lot like not knowing, you know, not being there with their animals.” This emotional impact created a dilemma for participants in pursuing veterinary services during the pandemic.

Furthermore, curbside service required veterinary clinics to adapt to virtual communication with companion animal guardians [75]. As explained above, certain (dis)abilities (e.g., sensory, cognitive, and motor) can reduce one’s capacity to use cell phone service [85]. Moreover, economic impacts resulted in some participants being unable to afford cell phone service. For example, one participant described how she arranged the use of a friend’s cell phone for her cat who required multiple visits: “So you go to the vet you drop your pet off, and then they call you on your phone while they’re doing the exam. Well, how am I going to get the message exactly? I don’t have a cell phone.” Hence, the adaptive veterinary service delivery model prevented this person from accessing veterinary service.

This accessibility challenge triggered more stress on companion animal guardians. Many participants deferred visitation to veterinary clinics unless it was an emergency.

One participant argued, “you know, if it’s an emergency, they’ll [veterinary clinic] just put them in, it’s, you know, it’s not like they need to come in for a nail trim like that can wait a few weeks.”

According to recent research, the emergency-only model has dramatically reduced the companion animal guardians’ inquiries for routine checks and vaccination [75]. The service reduction led some participants to pursue emergency care, accessing services from a new veterinarian where rapport has not been established, causing extra emotional stress for both companion animals and their guardians. One participant claimed, “I am worried a lot if my cat does not like the new vet.”

All the diverse impacts discussed above have jointly generated compounding feelings for companion animal guardians, including distrust, apprehension, and anxiety. These mixed feelings become a significant thrust for companion animal guardians to lean towards an urgent service-only model, which might influence companion animal welfare and companion animal guardians’ well-being.

## 5. Discussion

Affordability, feasibility, and accessibility are the three principal barriers identified in this research that jeopardize PWDs’ access to veterinary medical and behavioral services during the first lockdown of COVID-19. These three barriers reflect the ways this pandemic has largely overlooked PWDs’ unique requirements. The complexities associated with these barriers illustrate the root causes of social injustice that intensified during the pandemic. These barriers and complexities further generate new knowledge regarding improving these injustices through better meeting PWDs’ needs.

### 5.1. Visible Challenges and Invisible Health and Well-Being Influence

Affordability: The pre-COVID enduring barriers (low income and lack of financial support) and COVID-19-specific influences led to PWDs’ visible economic challenge of affordability and invisible emotional stress. Consequently, participants have pursued different sources of income and support, meeting their needs and their companion animals’ basic living needs (e.g., food and supplies) while living in a costly city. Participants in this study remain resourceful and creative in order to seek additional income based on their capacities, requesting financial support from their personal and social networks (e.g., family and friends). Under this economic hardship, when the need for veterinary medical care occurred, being unable to provide essential health services for their companion animals worsened these participants’ already precarious mental health conditions. Addressing economic hardship would be the first step to support PWDs and their companion animals’ health and well-being.

Feasibility: Participants were challenged with the feasibility of obtaining animal assistance program information during the pandemic, as information dissemination has not mobilized within traditional, familiar information outlets, such as community centers and connections with other companion animal guardians within their neighborhoods, as was typical before the pandemic. Internet-based information collection may not always be feasible due to PWDs’ various challenges, including physical, economic, and cognitive [86]. These limitations also impact their processes of filling out applications for assistance programs. Furthermore, these limitations, plus the financial support administrative process, may delay their strategies to secure veterinarian services for their companion animals, risking the animals’ health and, in turn, increasing guardian stress. Hence, the feasibility of obtaining related social support proved to be stressful for participants, resulting in negative health and wellness outcomes for both participants and their companion animals.

Accessibility: Public health safety measures impeded the fluidity of accessing public transit and instigated the altered curbside veterinary services [87]. Participants expressed fear of contracting COVID-19 during public transit commutes, as their risk factors were high due to their chronic health concerns. Additionally, the reduced hours and capacity of public transit services extended the participants’ trips to veterinary clinics. During the prolonged trips with their companion animals, they might have to deal with the animals’ unfavorable behaviors, which led to extra emotional stress. Moreover, communication barriers (e.g., lack of virtual communication equipment and unavailable face-to-face communication with their veterinary professionals) and the inability to accompany their companion animals in the examination rooms increased the participants’ difficulties and willingness to utilize the curbside service. The gratitude participants expressed for their families and friends’ assistance, especially the emotional support during transportation and curbside service, reaffirmed the negative impact on their health and well-being.

### 5.2. PWD-Driven Service Approaches

Recognizing the evidence-based challenges of PWDs must stimulate government and non-government agencies to improve their existing assistance programs and/or develop new strategies to address PWDs’ unique requirements. PWDs’ economic hardship could be alleviated by anticipating their particular challenges. For instance, the barriers PWDs face in applying for COVID-19-specific financial support could be removed. Since companion animals support PWDs’ health and well-being, financial assistance might consider extra funding to cover the additional costs associated with PWDs and their companion animals’ basic living requirements. Furthermore, veterinary clinics may provide increased payment flexibility or other possible support to increase the affordability of veterinary services for PWDs. As an illustration, to improve PWD companion animal health and welfare, veterinarians could incorporate a small necessary supply provision (e.g., hygiene and grooming tools as well as a small first aid kit) as a function of their practice.

Promoting PWDs’ access to the appropriate assistance and support programs would reduce the feasibility barrier. In fact, participants in this study have identified some possible resources that would ameliorate information access (e.g., community centers, service agencies for PWDs, and veterinary clinics). Ideally, community-based service agencies could promote their cross-institutional cooperation by coordinating their different resources, networks, and connections for information dissemination and co-design collaborative programs to target PWDs’ unique requirements. Additionally, simplifying eligibility criteria and applications for PWDs, to some extent, could alleviate their economic and emotional stress and swiftly provide necessary services for their companion animals. Similarly, this cross-institutional cooperation would reduce some COVID-19-specific accessibility-related challenges. To be precise, neighbor-based support programs (e.g., carpool and rideshare) would enable PWDs to arrive at veterinary clinics safely and effectively. These PWD-driven approaches would ultimately establish a healthy human–animal bond.

### 5.3. Limitations

The findings provide rich details regarding the PWDs’ challenges; however, the limitations are noticeable. Past studies have discovered solid interconnections between demographic information (e.g., age, sex, gender, ethnicity, (dis)abilities) and societal status (e.g., social, economic, cultural, and health and well-being) [88]. Although age, gender, and ethnicity demographic factors were collected in this project, data analysis only applied a (dis)ability lens. Further, within the lens of (dis)abilities, this study did not specify (dis)ability type, including physical (dis)ability, visual impairment, mental health challenges, and intellectual (dis)ability. Thus, future research could explore how different types of (dis)ability inform companion animal guardians’ behaviors and attitudes regarding veterinary services.

The research team’s expertise in disaster and emergency management, social work, sociology, and animal protection is precisely aligned with the fields in which this research is rooted. However, the researchers’ individual and professional backgrounds dramatically influenced the data interpretation. Hence, a more comprehensive interpretation would contribute to a nuanced understanding of these demographic factors’ influence on the challenges people living with (dis)abilities experience in accessing veterinary services, and would reduce relative bias.

Furthermore, the participants were recruited from a candidate pool from VHS and received financial support from this organization-designated program. These participants come from urban communities in Metro Vancouver, Canada. Differences between rural and urban communities also dictate different approaches to veterinary service access, which were not addressed in this study. Moreover, (dis)ability was not included as one of the inclusion and exclusion criteria. This limited the sample size of PWDs. These factors in the recruitment process potentially excluded other eligible participants. Accordingly, the research outcomes might not be transferable to other communities in Canada and beyond. Notwithstanding, these limitations display future research orientations. Prospective studies may engage detailed demographic stratifications into participant recruitment and data analysis. With PWDs as an example, future research may extend to other vulnerable and marginalized populations (e.g., people experiencing homelessness, older adults, children and youth, and Indigenous peoples) to systematically explore their unique in-group challenges or through cross-group comparison. Better vulnerability reduction strategies can be developed through a more comprehensive understanding, contributing to a more diverse, equal, and inclusive society.

## 6. Conclusions

The global public health crisis of COVID-19 has triggered diverse influences on humans and co-inhabitants. From the perspective of PWDs, this study qualitatively identified the challenges companion animal guardians face in obtaining veterinary services during the first wave of the lockdown, three-fold. COVID-19 worsened their already precarious economic status, resulting in affordability barriers to veterinary medical and behavioral care for their companion animals. Difficulties in obtaining information relating to assistance programs and facilitating the assistance program applications reduced their feasibility to receive timely extra financial support. The health and emotional concerns during transportation to veterinary clinics and the utilization of the veterinary curbside service decreased their accessibility and reshaped their attitudes toward accessing veterinary service. These three barriers, which are associated with various inequalities, have compounding impacts on the health and well-being of both companion animals and their guardians.

With the availability of vaccines worldwide, international communities are continually entering into the post-COVID-19 recovery stage. Lessons learned from PWDs’ experience provide a valuable opportunity to redress inequalities. Enhancements to various social assistance and support programs may embed PWD-driven approaches to more effectively address their unique requirements, especially during the emergency response stage for disaster events. The improvement will not only better prepare and support PWDs for future disaster events, but will also illuminate other vulnerable and marginalized groups so their particular requirements could also be recognized and addressed. Consequently, future research could engage other demographic factors associated with the element of (dis)ability to further understand PWDs’ challenges, or explore other vulnerable and marginalized groups’ COVID-19-related difficulties. All these efforts will fundamentally promote the development of an equal, just, and inclusive society, for PWDs, companion animal guardians, other vulnerable and marginalized groups, and beyond.

## Figures and Tables

**Table 1:** Participants’ demographic composition.

Participant	Gender	Age Group ^1^		Ethnicity Background	(Dis)Ability Self-Identification	Ways of Obtaining Animal(s)	Years with the Animal(s)
		18–39	40–59				
1	F		•	Caucasian	Unknown	Adopted	8 years
2	F	•		Caucasian	Yes	Adopted	3 years
3	F		•	Caucasian	Yes	Adopted	13 years
4	F		•	Caucasian	Yes	Adopted	9 years
5	F		•	Indigenous	Yes	Adopted	9.5 years
6	F		•	Caucasian	Yes	Adopted	9 years
7	F		•	Caucasian	Yes	Adopted	12 years
8	F	•		Indigenous	Unknown	Adopted	6 years
9	M		•	Caucasian	Yes	Adopted	10+ years
10	F		•	Caucasian	Unknown	Adopted	11 years
11	F		•	Indigenous	Yes	Adopted	2 years
12	F	•		Caucasian	Unknown	Adopted	6 years

^1^ “•” indicates the participant’s age group when the interview was conducted.

## Data Availability

The data underlying this article cannot be shared publicly due to privacy and ethical concerns. The data will be shared upon reasonable request to the corresponding author. The funders had no role in the design of the study; in the collection, analyses, or interpretation of data; in the writing of the manuscript, or in the decision to publish the results.
